# Randomized controlled trial of an 8-week intervention combining self-care and hypnosis for post-treatment cancer patients: study protocol

**DOI:** 10.1186/s12885-018-5046-6

**Published:** 2018-11-15

**Authors:** Charlotte Grégoire, Marie-Elisabeth Faymonville, Audrey Vanhaudenhuyse, Vanessa Charland-Verville, Guy Jerusalem, Isabelle Bragard

**Affiliations:** 1 Public Health Department and Sensation and Perception Research Group, GIGA-Consciousness, University of Liège, Liège, Belgium; 2 Algology-Palliative Care Department, CHU Liège, and Sensation and Perception Research Group, GIGA-Consciousness, University of Liège, Liège, Belgium; 3 GIGA-Consciousness, Coma Science Group & Neurology Department, University and CHU of Liège, Liège, Belgium; 4 Medical Oncology Department, CHU Liège and University of Liège, Liège, Belgium

**Keywords:** Oncology, Group intervention, Hypnosis, Fatigue, Emotional distress

## Abstract

**Background:**

Cancer has a lot of consequences on patients’ quality of life (such as cancer-related fatigue (CRF), sleep difficulties and emotional distress) and on patients’ partners and their relationship, such as distress and communication difficulties. These consequences are undertreated, and interventions based on hypnosis often focus on breast cancer patients only. This paper describes the study protocol of a longitudinal randomized controlled trial aiming to assess the efficacy of an 8-week intervention combining hypnosis and self-care to improve cancer patients’ CRF, sleep and emotional distress and to indirectly improve their partners’ distress.

**Methods:**

A power analysis required a total sample of 88 patients. To test the efficacy of the intervention, results of the experimental group receiving the intervention will be compared to those of the control group. Data will be collected by questionnaires, relaxation tasks, an attentional bias task, and everyday life assessments measured at four different times: 1.) before inclusion in the study (baseline); 2.) after the intervention; and 3.) at 4- and 12-month follow-up. Partners’ symptoms will also be evaluated with questionnaires at the same measurement times.

**Discussion:**

There is a growing interest in alternative approaches (such as hypnosis) in addition to standard therapies in oncology settings. The results of this study should be useful for improving knowledge about long-term efficacy of hypnosis-based group interventions for CRF, sleep and distress among all types of cancer patients and their partners, and to better understand the mechanisms of emotional regulation in cancer patients through the attentional bias task.

**Trial registration:**

ClinicalTrials.gov (NCT03144154). Retrospectively registered on the 1st of May, 2017.

**Electronic supplementary material:**

The online version of this article (10.1186/s12885-018-5046-6) contains supplementary material, which is available to authorized users.

## Background

It is predicted that 40% of the population will be diagnosed with cancer at least once, which has a lot of consequences for them and their relatives [1, 2]. First, a meta-analysis showed that 46–99% of cancer patients experience cancer-related fatigue (CRF) [3], which can be defined as “a distressing persistent, subjective sense of physical, emotional and/or cognitive tiredness related to cancer or cancer treatment that is not proportional to recent activity and interferes with usual functioning” [4]. The burden of CRF is high for patients who must endure social, financial, and functional negative consequences associated with this symptom [3, 5]. Second, a large proportion of cancer patients suffer from emotional distress, defined as “an unpleasant experience of an emotional, psychological, social, or spiritual nature that interferes with the ability to cope with cancer treatment” [6–10]. Emotional distress negatively influences treatment adherence [6] and results [6, 11, 12], and patient’s general quality of life [6, 13]. Fear of cancer recurrence is also linked to emotional distress [14–16]. One explanatory process of this relationship could be the presence of an attentional bias toward threat, which can be defined as selective processing of threatening information; in this case it is referring to cancer-related information [17–20]. Third, cancer also affects patients’ relatives. Partners of cancer patients often become the main caregiver for their ill spouse [21, 22] and can feel distressed [23–25] and tired [26, 27]. Changes in couple’s communication, intimacy, and sharing of responsibilities has also been noted [28–30]. Patients’ emotional distress and CRF in addition to their partners’ burden can persist for years after the end of cancer treatments [5, 31–33].

Despite the prevalence of patients’ emotional distress and CRF, and the impact of cancer on relatives, these consequences are still underdiagnosed and undertreated by healthcare professionals [6, 7, 34]. Some studies have shown the positive impact of psychological interventions on patients’ emotional distress [34–36] and CRF [35–38]. Some alternative methods such as hypnosis-based interventions have also been tested. Hypnosis can be defined as “a procedure during which a health professional or researcher suggests that a patient or subject experience changes in sensations, perceptions, thoughts, or behaviour” [39]. Several studies have demonstrated the positive impact on various sides effects of cancer treatments such as CRF, sleep, and distress of hypnosis taught alone [36, 38, 40–45], or combined with self-care techniques [41, 46, 47]. Most of these studies focus on breast cancer patients. However, other cancers may have different adverse physical and psychological effects. For example, mortality rates vary according to the localisation of the tumour [48]. Additionally, some cancers such as prostate and gynaecological cancer directly impact the patients’ sexuality, because they alter or remove symbols of masculinity or femininity (e.g. erection, libido, fertility…) [49–53], with high impact on couple’s intimacy and communication [29, 54–56]. Another example relates to brain tumours, which can lead to cognitive deficits, aphasia, visual field defects, motor deficits and personality changes [57, 58]. All these adverse effects are specific to the type of cancer and may have specific impact on the psychological adaptation to the disease.

## Objectives

Our primary goal is to assess the efficacy of an 8-week group intervention combining hypnosis and self-care to improve fatigue in all types of cancer patients, after treatments. The secondary goals are to assess the efficacy of this intervention to improve other patients’ well-being-related variables (emotional distress (anxiety and depression), sleep, fear of cancer recurrence, psychological adjustment to the disease, emotional regulation skills, cognitive functioning, and physical activity). As the intervention will address various fields such as communication with relatives, we will also assess its impact on the couple’s dynamic, and on partner’s distress. Finally, the link between the decrease in patients’ distress and a possible attentional bias will be assessed in order to better understand the emotional regulatory mechanisms in cancer patients.

## Methods and design

### Design

**Fig. 1:**
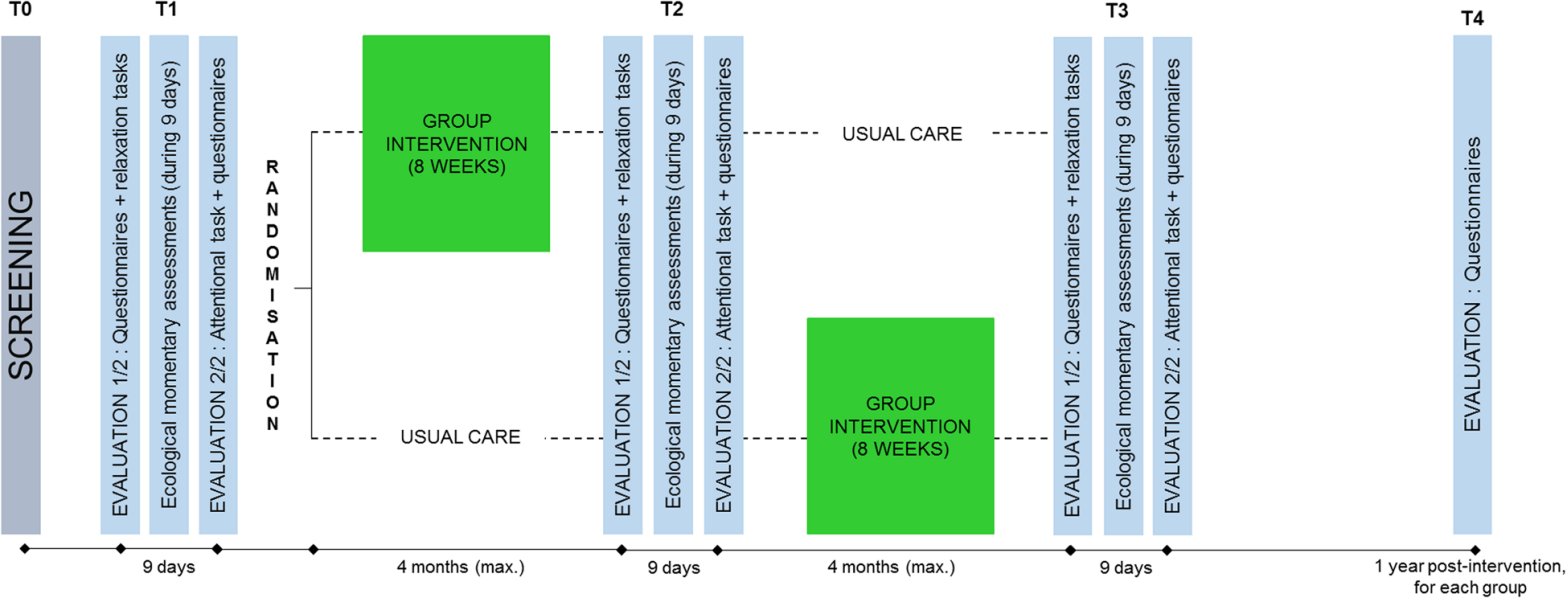
Design of the study

Figure 1 illustrates the longitudinal randomized waiting-list controlled trial design used in our study. Ethics approval has been obtained from the Comité d’Ethique Hospitalo-Facultaire Universitaire de Liège. The waiting-list control design allows every participant to benefit from the intervention, which will make it possible to recruit more patients than in a no-treatment control design. It also controls the influence of natural recovery from cancer treatments and spontaneous improvement or deterioration of symptoms [59]. Each intervention will start when 16 patients have successfully completed the screening and the first measurement time (T1). Those patients will be randomized into two groups of eight participants: the first group will receive immediate intervention (intervention group) and; the second group will receive it (at the latest) 4 months later (waiting-list control group).

The randomization process will be conducted by the principal investigator. Our clinical experience showed us that breast cancer patients are more likely to participate in such intervention. Despite the fact that we do not aim to compare the efficacy of our intervention between different cancer diagnoses, we will ensure that cancer diagnoses are equally allocated between the two groups (experimental vs control) because we want the same number of breast cancer patients in each of these groups. The researcher will conduct two randomizations: one for the breast cancer patients, and one for the other cancer patients. The draw will be operated through a specialized website (https://www.dcode.fr/tirage-au-sort-nombre-aleatoire) in which the investigator will introduce the codes of the participants and ask the software to divide them into two groups (experimental and control groups). The participants do not know the group in which they are included (experimental vs control) until the end of the first evaluation, when the experimenter tells them (T1). The first intervention group started in April 2017, and the waiting-list control group started in July 2017.

### Eligibility

Inclusion criteria include several parameters:

1. age 18 years or older;
2. fluent in French;
3. no history of psychiatric disorders, such as dementia, psychosis or delirium, which does not allow participation in a group intervention or completion of the evaluations;
4. diagnosis of non-metastatic cancer (all tumour localisations accepted); we did not include metastatic patients in order to minimize baseline differences in the sample, as the changing nature and complexity of metastatic cancers could make it difficult to compare the results with non-metastatic cases [60];
5. all active treatments completed for less than a year (surgery, chemotherapy, and/or radiotherapy), in order to form a group of participants who were recently involved in diagnosis and treatments and so increase the likelihood that the difficulties they are dealing with will be more similar;
6. no relapse at the time of inclusion;
7. difficulties as established by responses of at least 4 out of 10 on 1 of the 6 chosen items of the Edmonton Symptom Evaluation Scale [61]; these include physical fatigue, moral fatigue (we decided to split the original item “fatigue (lack of energy)” into two different items, in order to investigate both physical and psychological sides of fatigue), depression, anxiety, fear of recurrence, or ruminations. This cut-off score was chosen to avoid floor effects [59].

### Recruitment

Participants will be recruited over a 2-year period at the University Hospital of Liège (Belgium). Potentially eligible participants are identified in several ways. First, healthcare professionals working with cancer patients (mostly oncologists, radiotherapists, psychologists, and nurses) are informed of the study and asked to talk about it to their non-metastatic patients who have finished their treatment. Interested patients should directly report to the experimenter or are advised to contact us by phone. Second, flyers and posters are displayed in different strategic areas in the hospital (mostly in oncology, radiotherapy, and algology services’ waiting rooms), which allow other health professionals and patients to be informed about the study and to contact us to participate or to talk about the study. Finally, in collaboration with the social nurse, all eligible patients are directly met after their last radiotherapy session, and are offered to participate in the study. Review of the trial process and difficulties encountered are discussed once a month between the researchers. This permitted to improve our recruitment strategies.

The recruitment started in December 2016 and is ongoing. Sample size has been determined by a power analysis in order to detect a difference in the evolution of data between the two groups. The sample size calculation was based on an independent samples t-test for a difference in mean scores. Alpha was set at 0.05, power at 90% and the standardized effect size at 0.7. The Cohen’s *d* of 0.7 is considered to be a medium to high effect size. Psycho-oncology studies which aim to assess the effect of an intervention on the patients’ quality of life often use effect sizes between 0.6 and 0.8 to calculate the sample size [62, 63]. According to this analysis, 44 patients are required in each group for a total of 88 patients.

### Procedures

A written consent is obtained by the experimenter from each participant at the beginning of the study.

#### Screening (T0)

During the first telephone contact with the interested participants, they are informed of the protocol and study design. All participants are informed about study procedures and in particular, that they will receive the intervention immediately or after 4 months, according to the group they will be assigned to. If interested, the researcher ensures that participants meet all the inclusion criteria. If eligible, two appointments for the first evaluation (T1) are scheduled.

#### Measurement points (T1, T2 and T3)

Each of these three measurement points is completed by every participant and is divided into two sessions, separated by a 9-day interval (see Fig. 1). We decided to divide into two sessions in order to minimize the burden encountered by the participants. The first session (about 2 h 30 min) is dedicated to the completion of several questionnaires and the realization of two relaxation exercises (see Table 1). During the whole session, a cardiac holter monitor is placed on participants to measure their heart rate. The second session (about 1 h 15 min) is dedicated to a computerized attentional task and to the completion of some questionnaires and open questions (see Table 1). During the 9-day interval, participants wear an actigraph (Garmin Vivoactive® HR) which measures heart rate, physical activity, and sleep. As participants do not have to do anything else with the actigraph than to recharge it, it is not considered to be complicated. The experimenter also installs an application on their smartphone (RealLife Exp, pack LifeData V5), which asks them about their emotions six times a day. In Belgium, 64% of 45–54-year-old use a smartphone, while 53% of people aged of 55 or more use one [64]. We conclude, therefore that it is likely that most of our participants are able to use a smartphone. However, a smartphone is lent to those who don’t have one and explanations are given if necessary. The experimenter also remains available by phone or email between the sessions to answer any question. Some questionnaires for partners are also given to the participants who have one with instructions to ask their spouse to complete them in order to collect data about partners’ well-being. Consent forms for partners are attached to these questionnaires. At the end of the first measurement point, participants are informed of the result of the randomization and their allocation in either the intervention group or the waiting-list control group.

**Table 1:** Patients’ measures used in the study

T0 Screening	T1, T2 and T3 Measurement points	T4 Measurement point
*Questionnaires:*	Session 1	*Questionnaires:*
- ESAS (only 6 items)	*Questionnaires* - General information - VAS (psychological state) - ESAS (complete version) - HADS - PSWQ - FCRI - MAC - WBSI - MFI-20 - CERQ - ICQ - ISI	- MFI-20 - HADS - ESAS (complete version) - FCRI - Long-term benefits of the intervention
	*Tasks*	
	- Self-relaxation task + Questionnaire about relaxation strategies - Guided relaxation task + Questionnaire about relaxation habits	
	Between session 1 and session 2 (during 9 days) - Smartphone application (RealLifeExp) to collect information about daily emotional regulation. - Actigraph to measure sleep, activity and heart rate.	
	Session 2	
	*Questionnaires*	
	- VAS (motivation and implication) - Attentional bias task - FACT-Cog - FFMQ - MCQ-30 - PTGI - Rosenberg’s self-esteem scale - CICS - DCI - Expected/perceived benefits from the intervention	

*Abbreviations*: *VAS* Visual Analogue Scales, *ESAS* Edmonton Symptom Assessment Scale, *HADS* Hospital Anxiety and Depression Scale, *PSWQ* Penn State Worry Questionnaire, *FCRI* Fear of Cancer Recurrence Inventory, *MAC* Mental Adjustment to Cancer Scale, *WBSI* White Bear Suppression Inventory, *MFI-20* Multidimensional Fatigue Inventory, *CERQ* Cognitive Emotion Regulation Questionnaire, *ICQ* Impact of Cancer Questionnaire, *ISI* Insomnia Severity Index, *FACT-Cog* Functional Assessment of Cancer Therapy – Cognitive Function, *FFMQ* Five Facets Mindfulness Questionnaire, *MCQ-30* Metacognition Questionnaire, *PTGI* Post-Traumatic Growth Inventory, *CICS* Couples’ Illness Communication Scale, *DCI*, Dyadic Coping Inventory

#### One-year follow-up (T4)

One year after having participated in the intervention, each patient is contacted by the researcher to schedule a final group session with the therapist in order to evaluate the long-term benefits of the intervention. They discuss the application of learned skills in their daily life, perform one hypnosis exercise, and participants also have to complete some questionnaires (see Table 1). We expect the follow-up participation rate to be high, as most patient really appreciate the group session.

At each measurement point, the risk of missing responses is very low, as patients complete the scales in the presence of the investigator, who check every questionnaire after completion. All data are anonymized. A code is attributed to each participant and used during the whole study. Only the researchers involved in this study have access to the final datasets.

### Intervention

The intervention combining self-care and hypnosis includes eight weekly 2-h sessions in group of eight participants. This has been developed and is being led by one of the authors (MEF), who is an anaesthetist and an international expert in hypnosis. She introduced the use of hypnosis as an alternative for anaesthesia in surgery in our hospital in 1991, and has been the head of the continuing and palliative care unit since 2010, where she’s supported cancer patients for decades, basing her approach on self-care tasks [65]. She has led self-hypnosis and self-care groups since 2008 for chronic pain patients, and since 2013 for cancer patients.

The self-care approach is used to foster decision-making through the use of different tasks focused on well-being rather than on the disease itself. Participants have to complete these assigned tasks at home between sessions and keep a work-related diary to report how they managed it in their daily life. Examples of assignments are: adjusting self-expectation, reinforcing sense of self-esteem, revising self-narrative, adaptation of social roles, finding one’s own boundaries and personal needs, assertiveness, identifying situations and feeling of powerlessness, accepting that not everything is controllable, differentiating self from illness, managing ruminations, etc. These tasks illustrated with metaphors and humoristic anecdotes are given to patients in a practical and didactic way. This approach is based on self-management and patient empowerment approaches, which aim to strengthen self-esteem, assertion and self-confidence. Patients are encouraged to observe their thoughts and acts, and the different tasks proposed during and between sessions help them to detect and react to difficult situations. In this way, patients learn to adopt concrete changes aiming to respect themselves and others. They also learn to focus attention to positive things, life events that are going well, and to be grateful for “being alive”. An interactive and positive virtuous circle is constructed with patients all along sessions [47, 66]. Patients are asked to be actively involved in the process since the aim is to introduce change in their daily routines. In many ways, our strategies are similar with those developed in the cognitive-behavioural therapy (CBT) which is a “time-sensitive, structured, present-oriented psychotherapy directed toward solving current problems and teaching clients skills to modify dysfunctional thinking and behaviour” and which is “based on the cognitive model: the way that individuals perceive a situation is more closely connected to their reaction than the situation itself.” [67]. However, our intervention does not use CBT techniques such as cognitive restructuration or functional analysis by analysing a specific situation to understand its origin, but more generally to apprehend the future.

Concerning hypnosis, a large part of the first session is devoted to answering the participants’ questions and giving information about hypnosis. At the end of each session, a 15-min hypnosis exercise is conducted under the therapist’s supervision. Participants receive a CD for each exercise to encourage at-home practice, which is essential to take full advantage of hypnosis without the help of a therapist. It is attended that the practice of self-hypnosis will influence cognition and emotional regulation and therefore facilitate the completion of the assigned tasks. In this way, self-hypnosis is complementary to self-care tasks.

During the whole study duration, every participant in each group benefits from usual care, including medical care, oncological revalidation and individual psychological help if necessary. Although no adverse event has been reported in our previous studies [46, 47, 68], it could be possible that a patient feels uncomfortable during the group discussions or hypnosis exercises. In this case, they always have the possibility to stop the session or their participation in the study. The therapist or the experimenter can also propose a meeting to discuss their difficulties and, if necessary suggest a meeting with a psychologist or another health professional. Any reason for drop-out will be consigned.

### Assessments

Table 1 displays the different parameters used at each measurement time. They are detailed below.

#### Questionnaires

##### For patients

All questionnaires are detailed in Additional file 1: Appendix 1.

**General information:** Sociodemographic and medical data such as gender, age, language, professional activity, family members, personal history of cancer and treatment are collected. Some questions are also asked about life habits (physical activity, medication, alcohol and drug use, for example), and important life events.

**Physical and psychological functioning** is assessed through the use of several questionnaires: Visual Analogue Scales (VAS), the Edmonton Symptom Assessment Scale (ESAS) [61], the Hospital Anxiety and Depression Scale (HADS) [69], the Penn State Worry Questionnaire (PSQW) [70], the Fear of Cancer Recurrence Inventory (FCRI) [71], the Mental Adjustment to Cancer Scale (MAC) [72], the White Bear Suppression Inventory (WBSI) [73], the Multidimensional Fatigue Inventory (MFI-20) [74], the Cognitive Emotion Regulation Questionnaire (CERQ) [75], the Impact of Cancer Questionnaire (ICQ) [76], the Insomnia Severity Index (ISI) [77], the Five Facets Mindfulness Questionnaire (FFMQ) [78], the Post-traumatic Growth Inventory (PTGI) [79] and the Rosenberg’s self-esteem scale [80].

**Cognitive functioning** is assessed with the Functional Assessment of Cancer Therapy – Cognitive Function (FACT-Cog v.3) [81] and the Metacognition Questionnaire (MCQ-30) [82].

**Conjugal functioning** is assessed using two questionnaires: the Couples’ Illness Communication Scale (CICS) [83] and the Dyadic Coping Inventory (DCI) [84].

**Relaxation strategies** is investigated using two questionnaires about relaxation strategies used during the first exercise and about relaxation habits. These were created for the study.

Finally, the **questionnaire about the intervention** is focused on expected and perceived benefits from the intervention both for oneself and the partner (open questions).

##### For partners

Partners complete five questionnaires:

- *Sociodemographic and medical information*
- *Hospital Anxiety and Depression Scale* [69]
- *Couples’ Illness Communication Scale (CICS)* [83]
- *Dyadic Coping Inventory (DCI)* [84]
- Expected and perceived benefits from the intervention, for oneself and for the patient.

#### Tasks

Three tasks are suggested to the participants:

- *Two relaxation exercises:* Anxiety regulation is measured during two relaxation exercises. Before each exercise, anxiety is triggered by the completion of the MAC (before exercise 1) and the ICQ (before exercise 2) questionnaires. During the first exercise, participants have to use their own strategies to relax by themselves for about 13 mins, and during the second, they are guided by an audio recording for approximately the same time. Heart rate (beats per minute) is measured during the exercises by the Lifecard CF holter monitor as an indicator of anxiety regulation [85]. Heart rate variability has been shown to be correlated with emotion regulation [86] and influenced by emotional distress (depression and anxiety) [87, 88].
- *An attentional task:* A computerized task is used to assess the attentional bias toward threat. This computerized task, designed by the Université Libre de Bruxelles (ULB, Belgium), aims to evaluate the intensity of the attentional bias toward emotional information. Several pairs of words (each word being positive, negative, or neutral; and related or not related to cancer) are presented to the participants, followed by a point, located at the same place as one of the two words. The participants have to click on the up or down button of the keyboard depending of the location of the point. An increased response time for points following cancer-related words could suggest the presence of an attentional bias towards threat. Response time and accuracy will be measured in order to calculate different attentional bias indexes [18], which will evaluate the severity of the bias before and after the intervention.

#### Ecological momentary assessments

Different ecological momentary assessments will be made between the two evaluation sessions with the experimenter.

- *Emotional regulation:* At each measurement point, participants have to use an application on a smartphone for 9 days. This application was designed by ULB and aims to evaluate the emotional state of the participant at six different times of the day (intensity and perceived controllability of emotions, and energy level).
- *Activity and sleep measurements*: At each measurement point, participants wear an actigraph (Garmin Vivoactive® HR), which measures their physical activity (number of steps per day) and their sleep (number of hours of deep and light sleep per night and waking time after initial sleep onset) for 9 days. Wrist actigraphs provide an accurate 24-h sleep assessment and activity patterns in a natural environment [89, 90] and have been used in several studies with cancer patients [90–92].

### Data coding and storage

Most questionnaires are completed electronically (on a computer) by the participants. Data encoding will be assured by an independent team, in an automatic computerized way. They have no contact with the patients. Final databases, and manually coded data (data from the actigraphs, partners’ questionnaires) are stored on the experimenter’s computer, protected by a password.

### Statistical analyses

Baseline (T1) demographic, medical, and psychological data will be compared between groups to test for initial groups equivalency using inferential statistics, including analyses of variance (ANOVA) and chi-square tests. Group-by time changes, and pre- and post-assessment comparison of each variable within each group will be assessed using repeated measures MANOVA, on the participant who completed all the needed assessments times. The link between a decrease in distress and attentional bias will be assessed by correlations. Correlations will also be used to assess the links between the data from the patients and their partners, and we will measure the evolution of the partners using repeated measures MANOVA. All tests will be two-tailed, and the alpha will be set at 0.05. Statistical analyses will be performed after T3 and after T4.

All participants will be informed by e-mail about the final results of the study. Scientific publications will also be planned.

## Discussion

Few studies in oncology focus on CRF in cancer patients and interventions investigated in such studies are often traditional ones [37, 38]. Hypnosis-based interventions are starting to be evaluated as a way to improve quality of life as well [36, 40, 42]. However, long-term data about the effects of such interventions are missing, most studies included only breast cancer patients, and very few included partners in their assessments. Finally, very few studies have investigated the explanatory mechanisms of emotion regulation in cancer patients.

Thereby, our study would make a great contribution as it focuses on CRF, sleep, and emotional distress [6, 7, 34], and provide a long-term assessment of intervention effects. The randomised controlled design is also a strength of our study. Plus, it includes all types of cancer (except for metastatic ones) and all type of treatment, and evaluates the effects of the intervention on patients’ partners and on couple functioning. Finally, our study investigates the link between the attentional bias toward threat and emotion regulation, contributing to better understand the mechanisms of emotion regulation in cancer patients.

We made several hypotheses on the impact of the intervention: 1) fatigue and emotional distress will decrease in response to the intervention, and sleep and emotional regulation will improve; 2) dyadic coping and communication in the couple will improve, and partner’s distress will decrease; and 3) there will be a positive association between the decrease of patients’ distress and the decrease in the attentional bias.

These assumptions follow from the three major components of hypnosis that could influence cognition and emotional regulation: absorption, which is the involvement in a perceptual, imaginative or ideational experience; dissociation, which is the mental separation of different components of experience that would usually be processed as a whole; and suggestibility, which is the responsiveness to social clues, enhancing the propensity to comply with hypnotic instructions and suspending critical judgment [93]. Those hypnotic suggestions also seem to facilitate mind-body connection and lead to physical, emotional and behavioral changes [94].

There are some limitations of our study. The number and length of assessments completed by the participants could lead to some dropouts. It is possible that only motivated and compliant patients will complete all the assessments, which would bias the results of the study. However, it is important to point out that the participants have to complete assessments with at least a 3-month interval between them, which reduces the possible burden experienced by the participants. Another limitation is the fact that our intervention has only been proposed for non-metastatic patients in order to minimize the baseline differences of the sample.

In conclusion, our study will provide the initial insight into the short and long-term effects of an intervention combining hypnosis and self-care on the quality of life of all types of cancer patients in remission, on partners’ well-being, on the quality of the conjugal relationship, and on links between emotional regulation, distress, and attentional bias.

## Additional file

Additional file 1: Appendix 1. Questionnaires used in the study. Description of the questionnaires used in the study. (DOCX 25 kb)

## Abbreviations

CBT: Cognitive-Behavioural Therapy
CERQ: Cognitive Emotion Regulation Questionnaire
CICS: Couples’ Illness Communication Scale
CRF: Cancer-Related Fatigue
DCI: Dyadic Coping Inventory
ESAS: Edmonton Symptom Assessment Scale
FACT-Cog: Functional Assessment of Cancer Therapy – Cognitive Function
FCRI: Fear of Cancer Recurrence Inventory
FFMQ: Five Facets Mindfulness Questionnaire
HADS: Hospital Anxiety and Depression Scale
ICQ: Impact of Cancer Questionnaire
ISI: Insomnia Severity Index
MAC: Mental Adjustment to Cancer Scale
MCQ-30: Metacognition Questionnaire
MFI-20: Multidimensional Fatigue Inventory
PSWQ: Penn State Worry Questionnaire
PTGI: Post-Traumatic Growth Inventory
ULB: Université Libre de Bruxelles
VAS: Visual Analogue Scales
WBSI: White Bear Suppression Inventory

## References

[1] Ferlay J, Shin H-R, Bray F, Forman D, Mathers C, Parkin DM (2010). Estimates of worldwide burden of cancer in 2008: GLOBOCAN 2008. Int J Cancer.

[2] Ries L, Harkins D, Krapcho M, Mariotto A, Miller B, Feuer E (2006). SEER Cancer Statistics Review, 1975-2003.

[3] Prue G, Rankin J, Allen J, Gracey J, Cramp F (2006). Cancer-related fatigue: A critical appraisal. Eur. J. Cancer Oxf. Engl. 1990.

[4] Mock V, Atkinson A, Barsevick AM, Berger AM, Cimprich B, Eisenberger MA (2007). Cancer-related fatigue. Clinical practice guidelines in oncology. J Natl Compr Cancer Netw JNCCN.

[5] Jones JM, Olson K, Catton P, Catton CN, Fleshner NE, Krzyzanowska MK (2016). Cancer-related fatigue and associated disability in post-treatment cancer survivors. J Cancer Surviv.

[6] Dauchy S, Dolbeault S, Reich M (2013). Depression in cancer patients. EJC Suppl.

[7] Die TM (2013). Anxiety and sleep disorders in cancer patients. EJC Suppl.

[8] Hernández Blázquez M, Cruzado JA (2016). A longitudinal study on anxiety, depressive and adjustment disorder, suicide ideation and symptoms of emotional distress in patients with cancer undergoing radiotherapy. J Psychosom Res.

[9] Mitchell AJ, Chan M, Bhatti H, Halton M, Grassi L, Johansen C (2011). Prevalence of depression, anxiety, and adjustment disorder in oncological, haematological, and palliative-care settings: a meta-analysis of 94 interview-based studies. Lancet Oncol.

[10] NCCN practice guidelines for the management of psychosocial distress. National Comprehensive Cancer Network. Oncol. Williston Park N. 1999;13:113–47. 10370925

[11] Batty GD, Russ TC, Stamatakis E, Kivimäki M (2017). Psychological distress in relation to site specific cancer mortality: pooling of unpublished data from 16 prospective cohort studies. BMJ.

[12] Satin JR, Linden W, Phillips MJ (2009). Depression as a predictor of disease progression and mortality in cancer patients. Cancer.

[13] Achimas-Cadariu P, Iancu M, Pop F, Vlad C, Irimie A (2015). Psychological screening and health related quality of life in Romanian breast cancer survivors. J Evid-Based Psychother.

[14] Bridou M, Aguerre C (2010). Spécificités, déterminants et impacts de l’anxiété liée au cancer: revue de question des apports de l’approche cognitivo-comportementale. Psycho-Oncol..

[15] Lebel S, Rosberger Z, Edgar L, Devins GM (2009). Emotional distress impacts fear of the future among breast cancer survivors not the reverse. J Cancer Surviv Res Pract.

[16] Llewellyn CD, Weinman J, McGurk M, Humphris G (2008). Can we predict which head and neck cancer survivors develop fears of recurrence?. J Psychosom Res.

[17] Bardel M-H, Colombel F (2009). Rôles spécifiques de l’anxiété trait et état dans l’apparition et le maintien des biais attentionnels associés à l’anxiété : état des lieux et pistes d’investigation. L'Encéphale.

[18] Butow P, Kelly S, Thewes B, Hruby G, Sharpe L, Beith J (2015). Attentional bias and metacognitions in cancer survivors with high fear of cancer recurrence. Psychooncology.

[19] Custers JA, Becker ES, MFM G, HWM VL, Rinck M, Prins JB (2015). Selective attention and fear of cancer recurrence in breast cancer survivors. Ann Behav Med Publ Soc Behav Med.

[20] DiBonaventura MD, Erblich J, Sloan RP, Bovbjerg DH (2010). A computerized Stroop task to assess cancer-related cognitive biases. Behav Med Wash DC.

[21] Braun M, Mikulincer M, Rydall A, Walsh A, Rodin G (2007). Hidden morbidity in cancer: spouse caregivers. J. Clin. Oncol.

[22] Libert Y, Merckaert I, Étienne A-M, Farvacques C, Liénard A, Messin S (2006). Les besoins psychosociaux et le soutien apporté aux patients atteints d’un cancer: une étude nationale belge. Oncologie.

[23] Lapid MI, Atherton PJ, Kung S, Sloan JA, Shahi V, Clark MM (2016). Cancer caregiver quality of life: need for targeted intervention. Psychooncology.

[24] Schmid-Büchi S, van den Borne B, Dassen T, Halfens RJ (2011). Factors associated with psychosocial needs of close relatives of women under treatment for breast cancer: needs of relatives of women with breast cancer. J Clin Nurs.

[25] McClure KS, Nezu AM, Nezu CM, O’Hea EL, McMahon C (2012). Social problem solving and depression in couples coping with cancer. Psychooncology.

[26] Lee KC, Yiin JJ, Lin PC, Lu SH, Lee K-C, Yiin J-J (2015). Sleep disturbances and related factors among family caregivers of patients with advanced cancer. Psychooncology.

[27] Clark MM, Atherton PJ, Lapid MI, Rausch SM, Frost MH, Cheville AL (2014). Caregivers of patients with Cancer fatigue: a high level of symptom burden. Am J Hosp Palliat Med.

[28] Fletcher PC (2010). My child has Cancer: the costs of mothers’ experiences of having a child with pediatric Cancer. Issues Compr Pediatr Nurs.

[29] Goldsmith DJ, Miller GA (2014). Conceptualizing how couples talk about Cancer. Health Commun.

[30] Wittmann D, Carolan M, Given B, Skolarus TA, An L, Palapattu G (2014). Exploring the role of the partner in couples’ sexual recovery after surgery for prostate cancer. Support. Care Cancer off. J. Multinatl. Assoc. support. Care Cancer..

[31] Holden AEC, Ramirez AG, Gallion K (2014). Depressive symptoms in Latina breast cancer survivors: a barrier to cancer screening. Health Psychol. Off. J. Div. Health Psychol. Am. Psychol. Assoc.

[32] Wang XS, Zhao F, Fisch MJ, O’Mara AM, Cella D, Mendoza TR (2014). Prevalence and characteristics of moderate to severe fatigue: a multicenter study in cancer patients and survivors. Cancer.

[33] Beesley VL, Price MA, Webb PM, Australian ovarian Cancer study group, Australian Ovarian Cancer Study—Quality of Life Study Investigators (2011). Loss of lifestyle: health behaviour and weight changes after becoming a caregiver of a family member diagnosed with ovarian cancer. Support Care Cancer.

[34] de Vries M, Stiefel F, Goerling U (2014). Psycho-Oncological Interventions and Psychotherapy in the Oncology Setting. Psychooncology.

[35] Björneklett HG, Rosenblad A, Lindemalm C, Ojutkangas M-L, Letocha H, Strang P (2013). A randomized controlled trial of support group intervention after breast cancer treatment: results on sick leave, health care utilization and health economy. Acta Oncol Stockh Swed.

[36] Bragard I, Etienne A-M, Faymonville M-E, Coucke P, Lifrange E, Schroeder H, et al. A non-randomized comparison study of self-hypnosis, yoga and cognitive behavioral therapy to reduce emotional distress in breast cancer patients. Int J Clin Exp Hypn. 2017; Available from: http://orbi.ulg.ac.be/handle/2268/195981. [cited 13 Jan 2017]. 10.1080/00207144.2017.1276363 28230462

[37] Campos MPO, Hassan BJ, Riechelmann R, Del Giglio A (2011). Cancer-related fatigue: a review. Rev Assoc Medica Bras. 1992.

[38] Mitchell SA, Hoffman AJ, Clark JC, DeGennaro RM, Poirier P, Robinson CB (2014). Putting evidence into practice: an update of evidence-based interventions for Cancer-related fatigue during and following treatment. Clin J Oncol Nurs.

[39] The Executive Committee of the American Psychological Association (1994). Division of Psychological Hypnosis. Definition and description of hypnosis. Contemp Hypn.

[40] Montgomery GH, Schnur JB, Kravits K (2013). Hypnosis for cancer care: over 200 years young. CA Cancer J Clin.

[41] Charland-Verville V, Faymonville M-E, Vanhaudenhuyse A, Raaf M, Grégoire C, Bragard I (2017). Apprentissage de l’autohypnose/autobienveillance en oncologie. Pour qui ? Comment ? Dans quel intérêt ? Une revue de la littérature internationale. Psycho-Oncol.

[42] Cramer H, Lauche R, Paul A, Langhorst J, Kümmel S, Dobos GJ (2015). Hypnosis in breast cancer care: a systematic review of randomized controlled trials. Integr Cancer Ther.

[43] Kwekkeboom KL, Cherwin CH, Lee JW, Wanta B (2010). Mind-body treatments for the pain-fatigue-sleep disturbance symptom cluster in persons with cancer. J Pain Symptom Manag.

[44] Mendoza ME, Capafons A, Gralow JR, Syrjala KL, Suárez-Rodríguez JM, Fann JR, et al. Randomized controlled trial of the Valencia model of waking hypnosis plus CBT for pain, fatigue, and sleep management in patients with cancer and cancer survivors: Valencia model of waking hypnosis for managing cancer-related symptoms. Psychooncology. 2016; Available from: http://doi.wiley.com/10.1002/pon.4232. [cited 2 Dec 2016]. 10.1002/pon.4232 PMC5865590 27467589

[45] Montgomery GH, Kangas M, David D, Hallquist MN, Green S, Bovbjerg DH (2009). Fatigue during breast cancer radiotherapy: an initial randomized study of cognitive–behavioral therapy plus hypnosis. Health Psychol.

[46] Grégoire Charlotte, Bragard Isabelle, Jerusalem Guy, Etienne Anne-Marie, Coucke Philippe, Dupuis Gilles, Lanctôt Dominique, Faymonville Marie-Elisabeth (2017). Group interventions to reduce emotional distress and fatigue in breast cancer patients: a 9-month follow-up pragmatic trial. British Journal of Cancer.

[47] Vanhaudenhuyse A, Gillet A, Malaise N, Salamun I, Barsics C, Grosdent S (2015). Efficacy and cost-effectiveness: a study of different treatment approaches in a tertiary pain Centre. Eur J Pain Lond Engl.

[48] Jemal A, Bray F, Center MM, Ferlay J, Ward E, Forman D (2011). Global cancer statistics. CA Cancer J Clin.

[49] Selli C, Bjartell A, Burgos J, Somerville M, Palacios J-M, Benjamin L (2014). Burden of illness in prostate Cancer patients with a low-to-moderate risk of progression: a one-year, Pan-European Observational Study. Prostate Cancer.

[50] Donovan KA, Walker LM, Wassersug RJ, Thompson LMA, Robinson JW (2015). Psychological effects of androgen-deprivation therapy on men with prostate cancer and their partners. Cancer.

[51] Park HY, Kim JH, Choi S, Kang E, Oh S, Kim JY (2015). Psychological effects of a cosmetic education programme in patients with breast cancer. J Cancer Care.

[52] Karabulut N, Erci B (2009). Sexual desire and satisfaction in sexual life affecting factors in breast cancer survivors after mastectomy. J Psychosoc Oncol.

[53] Giacomoni C, Venturini E, Hoarau H, Guyon F, Conri V (2014). How women with gynaecological cancer deal with treatment: issues of visibility and invisibility. Gynecol Obstet Fertil.

[54] Manne S, Badr H, Zaider T, Nelson C, Kissane D (2010). Cancer-related communication, relationship intimacy, and psychological distress among couples coping with localized prostate cancer. J Cancer Surviv.

[55] Carter N, Bryant-Lukosius D, DiCenso A, Blythe J, Neville AJ (2011). The supportive care needs of men with advanced prostate Cancer. Oncol Nurs Forum.

[56] Martin E, Bulsara C, Battaglini C, Hands B, Naumann FL (2015). Breast and prostate cancer survivor responses to group exercise and supportive group psychotherapy. J Psychosoc Oncol.

[57] Osoba D, Brada M, Prados MD, Yung WK (2000). Effect of disease burden on health-related quality of life in patients with malignant gliomas. Neuro-Oncol.

[58] Heimans JJ, Taphoorn MJB (2002). Impact of brain tumour treatment on quality of life. J Neurol.

[59] Zernicke KA, Campbell TS, Speca M, McCabe-Ruff K, Flowers S, Dirkse DA (2013). The eCALM trial-eTherapy for cancer appLying mindfulness: online mindfulness-based cancer recovery program for underserved individuals living with cancer in Alberta: protocol development for a randomized wait-list controlled clinical trial. BMC Complement Altern Med.

[60] Cardoso F, Costa A, Norton L, Cameron D, Cufer T, Fallowfield L (2012). 1st international consensus guidelines for advanced breast cancer (ABC 1). Breast Edinb Scotl.

[61] Chang VT, Hwang SS, Feuerman M (2000). Validation of the Edmonton symptom assessment scale. Cancer.

[62] Reid J, Scott D, Santin O, Cardwell CR, Donnelly M, Kernohan WG (2014). Evaluation of a psychoeducational intervention for patients with advanced Cancer who have Cachexia and their lay Carers (EPACaCC): study protocol. J Adv Nurs.

[63] van der Spek N, Vos J, van Uden-Kraan CF, Breitbart W, Cuijpers P, Knipscheer-Kuipers K (2014). Effectiveness and cost-effectiveness of meaning-centered group psychotherapy in cancer survivors: protocol of a randomized controlled trial. BMC Psychiatry.

[64] Centrum voor Informatie over de Media (CIM) (2017). Smartphone User Penetration in Belgium, by Age and Language, 2016.

[65] Faymonville M-E, Bejenke CJ, Hansen E. Hypnotic techniques. In: Cyna A, Andrew MI, Tan SGM, Smith AF, eds. Handbook of Communication in Anaesthesia and Critical Care. Oxford: Oxford University Press; 2010. p. 249–261.

[66] Vanhaudenhuyse A, Gillet A, Malaise N, Salamun I, Grosdent S, Maquet D, et al. Psychological interventions influence patients’ attitudes and beliefs about their chronic pain. J. Tradit. Complement. Med. 2017; Available from: http://orbi.ulg.ac.be/handle/2268/207607. [cited 21 Nov 2017]. 10.1016/j.jtcme.2016.09.001 PMC5934699 29736385

[67] Beck Institute for Cognitive Behavior Therapy (2016). What is cognitive behavior therapy (CBT)?.

[68] Vanhaudenhuyse A, Faymonville M-E (2015). Intérêt de l’hypnose dans le domaine du soin. Rev Prat.

[69] Zigmond AS, Snaith RP (1983). The hospital anxiety and depression scale. Acta Psychiatr Scand.

[70] Meyer TJ, Miller ML, Metzger RL, Borkovec TD (1990). Development and validation of the Penn State worry questionnaire. Behav Res Ther.

[71] Simard S, Savard J (2009). Fear of Cancer recurrence inventory: development and initial validation of a multidimensional measure of fear of cancer recurrence. Support. Care Cancer.

[72] Watson M, Greer S, Young J, Inayat Q, Burgess C, Robertson B (1988). Development of a questionnaire measure of adjustment to cancer: the MAC scale. Psychol Med.

[73] Wegner DM, Zanakos S (1994). Chronic thought suppression. J Pers.

[74] Smets EM, Garssen B, Bonke B, De Haes JC (1995). The multidimensional fatigue inventory (MFI) psychometric qualities of an instrument to assess fatigue. J Psychosom Res.

[75] Garnefski N, Kraaij V, Spinhoven P (2001). Negative life events, cognitive emotion regulation and emotional problems. Personal Individ Differ.

[76] Zebrack BJ (2011). Psychological, social, and behavioral issues for young adults with cancer. Cancer.

[77] Savard M-H, Savard J, Simard S, Ivers H (2005). Empirical validation of the insomnia severity index in cancer patients. Psychooncology.

[78] Baer RA, Smith GT, Lykins E, Button D, Krietemeyer J, Sauer S (2008). Construct validity of the five facet mindfulness questionnaire in meditating and nonmeditating samples. Assessment.

[79] Tedeschi RG, Calhoun LG (1996). The posttraumatic growth inventory: measuring the positive legacy of trauma. J Trauma Stress.

[80] Rosenberg M. Conceiving the self. New York: Basic Books; 1979.

[81] Joly F, Lange M, Rigal O, Correia H, Giffard B, Beaumont JL (2012). French version of the functional assessment of Cancer therapy-cognitive function (FACT-cog) version 3. Support. Care Cancer.

[82] Wells A, Cartwright-Hatton S (2004). A short form of the metacognitions questionnaire: properties of the MCQ-30. Behav Res Ther.

[83] Arden-Close E, Moss-Morris R, Dennison L, Bayne L, Gidron Y (2010). The couples’ illness communication scale (CICS): development and evaluation of a brief measure assessing illness-related couple communication. Br J Health Psychol.

[84] Bodenmann G. Dyadisches coping Inventar: DCI; Manual. Bern: Huber; 2008.

[85] Merckaert I, Lewis F, Delevallez F, Herman S, Caillier M, Delvaux N, et al. Improving anxiety regulation in patients with breast cancer at the beginning of the survivorship period: a randomized clinical trial comparing the benefits of single-component and multiple-component group interventions: Improving anxiety regulation. Psychooncology. 2016; Available from: http://doi.wiley.com/10.1002/pon.4294. [cited 2 Dec 2016 ]. 10.1002/pon.4294 27718533

[86] Thayer JF, Ahs F, Fredrikson M, Sollers JJ, Wager TD (2012). A meta-analysis of heart rate variability and neuroimaging studies: implications for heart rate variability as a marker of stress and health. Neurosci Biobehav Rev.

[87] Bassett D (2016). A literature review of heart rate variability in depressive and bipolar disorders. Aust N Z J Psychiatry.

[88] Chalmers JA, Quintana DS, Abbott MJ-A, Kemp AH (2014). Anxiety disorders are associated with reduced heart rate variability: a meta-analysis. Front Psychiatry.

[89] Kripke DF, Hahn EK, Grizas AP, Wadiak KH, Loving RT, Poceta JS (2010). Wrist actigraphic scoring for sleep laboratory patients: algorithm development. J Sleep Res.

[90] Moore CM, Schmiege SJ, Matthews EE (2015). Actigraphy and sleep diary measurements in breast Cancer survivors: discrepancy in selected sleep parameters. Behav Sleep Med.

[91] Olson K (2014). Sleep-related disturbances among adolescents with cancer: a systematic review. Sleep Med.

[92] Kaleyias J, Manley P, Kothare SV (2012). Sleep disorders in children with cancer. Semin Pediatr Neurol.

[93] Vanhaudenhuyse A, Laureys S, Faymonville M-E (2014). Neurophysiology of hypnosis. Neurophysiol Clin Neurophysiol.

[94] Sawni Anju, Breuner Cora (2017). Clinical Hypnosis, an Effective Mind–Body Modality for Adolescents with Behavioral and Physical Complaints. Children.

