# Supplementary material for: Randomized controlled trial of an 8-week intervention combining self-care and hypnosis for post-treatment cancer patients: study protocol
Source: BMC Cancer. 2018 Nov 15;18:1113. doi: 10.1186/s12885-018-5046-6 (PMC6238378; doi:10.1186/s12885-018-5046-6)
Supplement: Supplementary file 1 — Appendix 1. Questionnaires used in the study. Description of the questionnaires used in the study. (DOCX 25 kb) [file 12885_2018_5046_MOESM1_ESM.docx]

**Appendix 1: questionnaires used in the study**

*Visual Analogue Scales (VAS):* Different VAS will assess the participants’ emotional state (at the moment and in general) in several dimensions: anxiety, sadness, fear of recurrence, physical fatigue, mental fatigue, energy, relaxation, ruminations…

*Edmonton Symptom Assessment Scale (ESAS)* (Chang, Hwang, & Feuerman, 2000): This 9-item visual analogue scale was developed to assess the symptoms (physical and emotional) of patients with cancer.

*Hospital Anxiety and Depression Scale (HADS)* (Zigmond & Snaith, 1983): This 14-item questionnaire is designed to measure anxiety (7 items) and depression (7 items). It has been validated for people with somatic illnesses.

*Penn State Worry Questionnaire (PSQW)* (Meyer, Miller, Metzger, & Borkovec, 1990): This 16-item instrument measures the level of worry of the participants.

*Fear of Cancer Recurrence Inventory (FCRI)* (Simard & Savard, 2009): This 42-item 5-point scale is composed of seven subscales: triggers, severity, psychological distress, coping strategies, functioning impairments, insight and reassurance.

*Mental Adjustment to Cancer Scale (MAC)* (Watson et al., 1988): This questionnaire is designed to assess the participant’s coping styles and adjustment to cancer and is divided into two sub-scales: positive adjustment and negative adjustment.

*White Bear Suppression Inventory (WBSI)* (Wegner & Zanakos, 1994): This 15-item scale measures thought suppression. This variable is related to negative affects and obsessive thinking, which can be associated with depression and anxiety.

*Multidimensional Fatigue Inventory (MFI-20)* (Smets, Garssen, Bonke, & De Haes, 1995): This 20-item questionnaire is designed to measure fatigue. It covers 5 different dimensions: general fatigue, physical fatigue, mental fatigue, reduced motivation and reduced activity.

*Cognitive Emotion Regulation Questionnaire (CERQ)* (Garnefski, Kraaij, & Spinhoven, 2001): This multidimensional questionnaire investigates the cognitive emotion regulation strategies used by the participant after experiencing negative events linked with the disease or its treatments.

*Impact of Cancer Questionnaire (ICQ)* (Zebrack, Ganz, Bernaards, Petersen, & Abraham, 2006): This 37-item scale is designed to assess the physical and psychological experience of cancer survivors through the positive and negative impacts of the disease.

*Insomnia Severity Index (ISI)* (Savard, Savard, Simard, & Ivers, 2005)*:* This 7-item scale investigates the participant’s sleep complaints and the distress associated.

*Five Facets Mindfulness Questionnaire (FFMQ)* (Baer et al., 2008): This questionnaire measures five components of mindfulness: observing, describing, acting with awareness, non-judging of inner experience and non-reactivity to inner experience.

*Post-traumatic Growth Inventory* (Tedeschi & Calhoun, 1996): This 21-item scale assesses positive outcomes reported after traumatic events. Five factors are investigated: new possibilities, relating to others, personal strength, spiritual change and appreciation of life.

*Rosenberg’s Self-Esteem Scale* (Rosenberg, 1979): This 10-item scale measures global self-esteem through positive and negative feelings about the self.

*Functional Assessment of Cancer Therapy – Cognitive Function (FACT-Cog v.3)* (Joly et al., 2012): This 20-item instrument measures the participant’s subjective cognitive functioning over the past week.

*Metacognition Questionnaire (MCQ-30)* (Wells & Cartwright-Hatton, 2004): This 30-item questionnaire evaluates the participant’s metacognitive beliefs, judgments and monitoring tendencies. Five factors are distinguished: cognitive confidence, positive beliefs about worry, cognitive self-consciousness, negative beliefs about uncontrollability of thoughts and danger, and beliefs about need to control thoughts.

*Couples’ Illness Communication Scale (CICS)* (Arden-Close, Moss-Morris, Dennison, Bayne, & Gidron, 2010): This 4-item scale measures the illness-related communication between partners. The French Translation of this scale was done by the author (CG).

*Dyadic Coping Inventory (DCI)* (Bodenmann, 2008): This 37-item instrument measures dyadic coping (supportive, delegated, negative and joint) occurring in conjugal relationships when one or both partners are stressed. Dyadic coping includes the attempts of a person to reduce their partner’s stress and the efforts made by the two partners to deal with external stressors that affect the relationship.

*Questionnaire about relaxation strategies* is designed to assess which strategies participant has used to relax during the self-relaxation exercise.

*Questionnaire about relaxation habits* is designed to assess the daily life relaxation practice.
